# Immunization Strategies against *Piscirickettsia salmonis* Infections: Review of Vaccination Approaches and Modalities and Their Associated Immune Response Profiles

**DOI:** 10.3389/fimmu.2016.00482

**Published:** 2016-11-18

**Authors:** Øystein Evensen

**Affiliations:** ^1^Faculty of Veterinary Medicine and Biosciences, Norwegian University of Life Sciences, Oslo, Norway

**Keywords:** *Piscirickettsia salmonis*, immunity, vaccination, modalities, efficacy

## Abstract

Salmonid rickettsial septicemia (SRS) is a serious, infectious disease in Chilean salmon farming caused by *Piscirickettsia salmonis*, causing heavy losses to the salmonid industry. *P. salmonis* belongs to the *Gammaproteobacteria*, order *Thiotrichales*. SRS was first described in Chile in 1989, and infection with *P. salmonis* has since been described from a high number of fish species and in several geographic regions globally. *P. salmonis* infection of salmonids causes multifocal, necrotic areas of internal organs such as liver, kidney, and spleen. Histologically and immunologically, the tissue response is the formation of granulomas, often with central suppuration. The exact sequence of infection is not known, but bacteria likely gain access to internal organs through mucosal surfaces and when infected, fish carry bacteria in macrophages. It has not been fully determined if the bacterium resides in the cytosol or “hide” within vesicular structures intracellularly, although there are indications that *in vitro* infection results in actin reorganization and formation of actin-coated vesicle within which the bacterium resides. Protection against lethal challenge is well documented in lab scale experiments, but protection from vaccination has proven more difficult to attain long term under field conditions. Current vaccination protocols include whole cell, inactivated and adjuvanted vaccines for injection for primary immunization followed by oral boost where timing of boost delivery is followed by measuring circulating antibody levels against the pathogen. Documentation also exist that there is correlation between antibody titers and protection against mortality. Future vaccination regimes will likely also include live-attenuated vaccines or other technologies such as DNA vaccination. So far, there is no documentation available for live vaccines and, for DNA vaccines, studies have been unsuccessful under laboratory conditions.

## Introduction

Salmonid rickettsial septicemia (SRS) was first observed in Chile in 1989 (1, 2), and the etiology of the disease was not understood at the time. It affected market-size Coho salmon (*Onchorhyncus kisutch*), and mortality was observed several weeks after transfer to seawater, reaching up to 90% (2). It was soon after documented that SRS was caused by infection with *Piscirickettsia salmonis* (3). Similar disease outbreaks have later been diagnosed in Ireland and Scotland (4), Norway (5), and the Atlantic and Pacific coasts of Canada (6). SRS still causes major losses in salmon farming in Chile, and current annual losses are estimated at 250 million USD and the infection results in a high consumption of antibiotics toward the end of the production cycle (7). Disease outbreaks are seen in all salmonid species farmed in Chile, Chinook salmon (*Oncorhynchus tshawytscha*), rainbow trout (*Oncorhynchus mykiss*), and Atlantic salmon (*Salmo salar* L.) (7), and also in other species like Sakura salmon (*Oncorhynchus masou*) (8), and pink salmon (*Oncorhynchus gorbuscha*) (1). Infections with *Rickettsia*-like organisms (RLO) have been reported in Mediterranean sea bass (*Dicentrarchus labrax*) (9), white sea bass (*Atractoscion nobilis*) (10), grouper (*Epinephelus melanostigma*) (11), and five species of tilapia (12). Globally, infections with piscirickettsia and RLO cause severe losses in farmed fish species (Table 1).

**Table 1:** **Overview of host range and geography of reported cases of piscirickettsiosis**.

Host	Geography	References
*Salmo salar* – Atlantisk laks	Canada – Atlantic ocean	(4, 5, 66, 67)
	Norway	
	Ireland	
	Scotland	
*Oncorhynchus gorbuscha –* Pink salmon	Canada – Pacific Ocean	(68)
*Oncorhynchus tshawytscha –* Chinook salmon		
*Salmo salar* – Atlantic salmon		
*Oncorhynchus kisutch –* Coho salmon	Chile	(2, 8, 69)
*Oncorhynchus mykiss –* rainbow trout		
*Oncorhynchus tshawytscha –* Chinook salmon		
*Salmo salar* – Atlantic salmon		
*Oncorhynchus masou –* Masu salmon		
*Atractoscion nobilis* – White sea bass	USA	(70)
*Epinephelus melanostigma* Grouper	Taiwan	(11)

## Etiology

*Piscirickettsia salmonis*, the causative agent of SRS is a Gram-negative, non-motile, non-encapsulated, 0.5–1.5 μm, intracellular bacterium (1, 13) that also grows *in vitro* in cell-free media (14–16). The bacterium is classified into a new family *Piscirickettsiaceae* in the phylum Proteobacteria, class *Gammaproteobacteria*, and order *Thiotrichales* (1). It was assigned to a new genus and species *P. salmonis* (13) with the type strain LF-89 (1, 17). It can infect a wide variety of cells lines, such as RTG-2, CHSE-214, RTS-11, and also Sf-21 cells (18), the latter being an insect cell line that yields high titer (19). The understanding is that *P. salmonis* replicates within membrane-bound cytoplasmic vacuoles by binary fission (12, 20), and *P. salmonis* survives and multiplies in macrophages (21). In Chile, the disease normally occurs 6–12 weeks after introduction to seawater, but it is seen throughout the production cycle, resulting in high losses of larger fish. Moribund fish appear dark, anorexic or lethargic, and swim near the surface or edges of the cage (1, 17). Some fish may also present skin lesions: hemorrhages, petecchiae, nodules, and ulcers of varying size (20). Brain infection also occurs and the bacterium is also able to form biofilm under given conditions (22).

## Pathology

**Figure 1:**
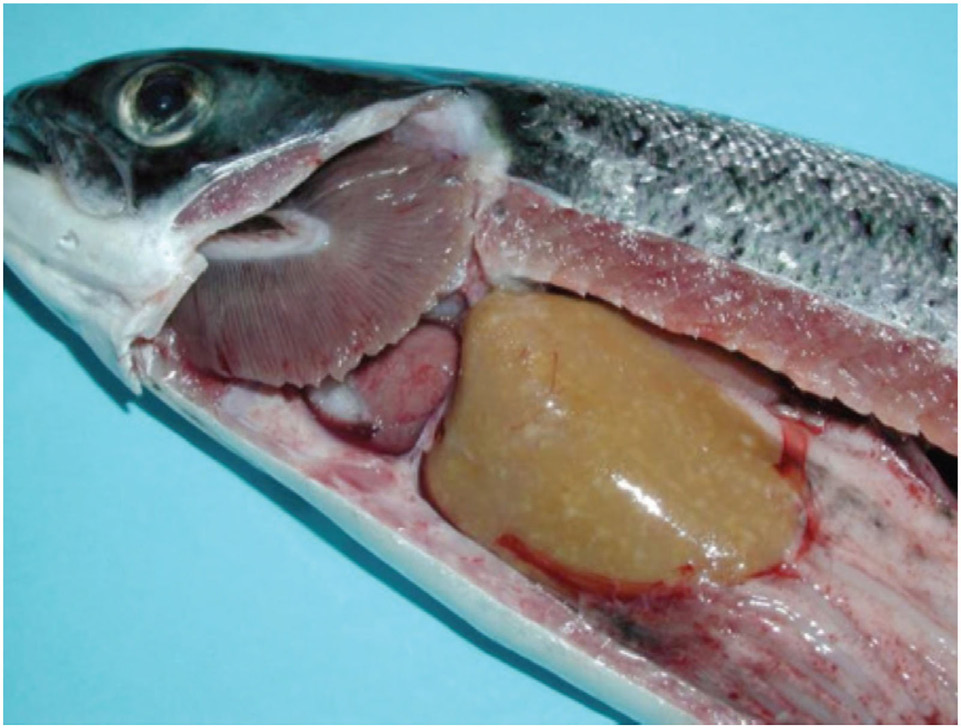
**Macroscopic changes in Atlantic salmon infected with *Piscrickettsia salmonis***. Note small, gray foci in the liver parenchyma of varying sizes. Gills are found with a grayish surface and the heart is pale (courtesy of Prof. Sandra Bravo, Universidad de Austral, Chile).

**Figure 2:**
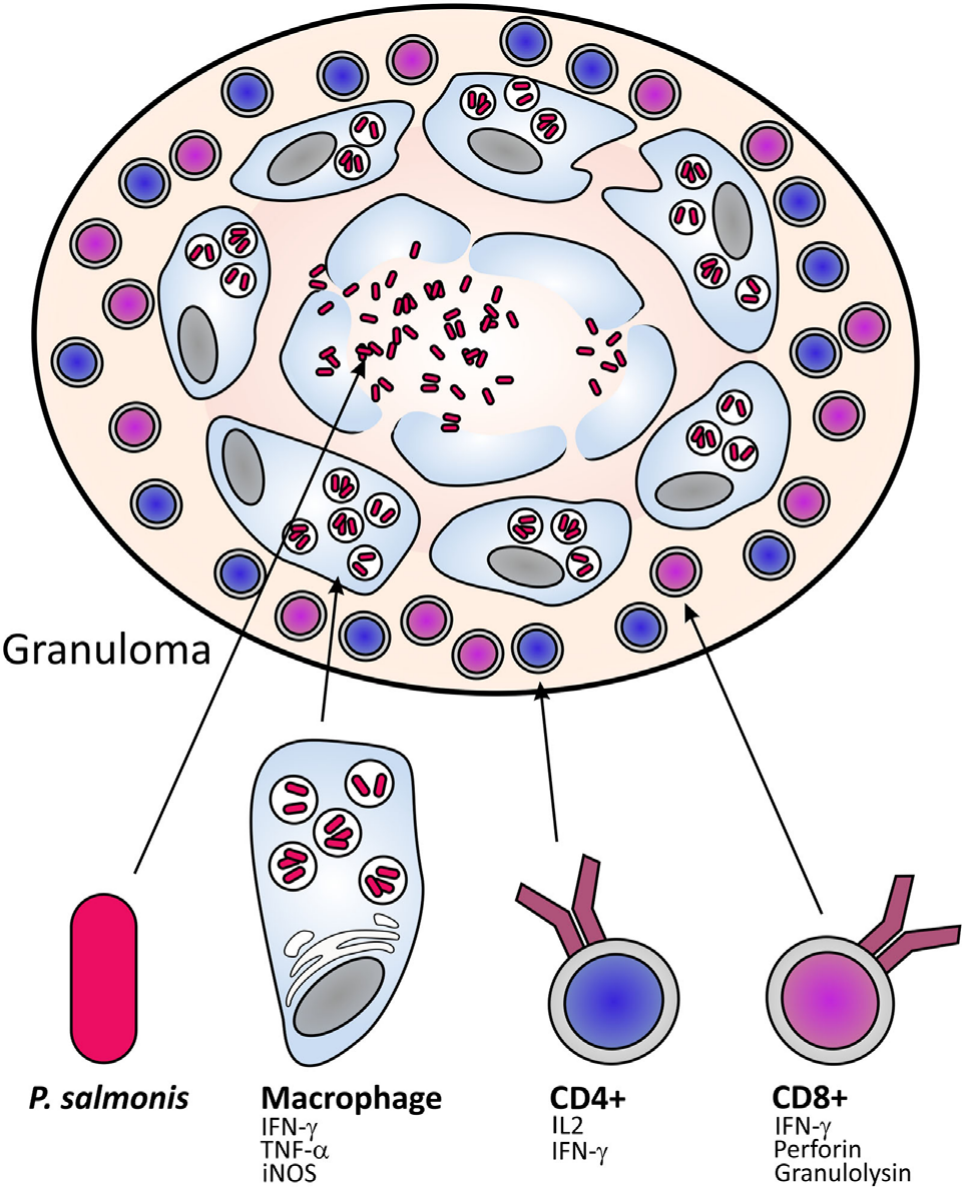
**Organization of granulomas**. In general, CD4 and CD8 cells play a role in the granulomatous response. CD4 cells activate the macrophages to enable them to kill the phagocytized pathogen, and CD8 cells will play a role in killing the invading pathogen, likely through killing of infected macrophages. The precise involvement of these cells in the response to *P. salmonis* infection is not understood.

Salmonid rickettsial septicemia in Atlantic salmon is often found with liver changes characterized by multifocal, necrotic areas of the hepatic parenchyma (Figure 1). Histologically, the typical tissue response to infection is the formation of granulomas, often with central suppuration and changes are seen in liver, spleen, and kidney (23), for this reason, the changes have been classified into the broad category of necrosis and inflammation but the principal changes are those of a granulomatous response that are more or less organized (5). At early stage of infection, granulomas typically consist of macrophages and a large number of neutrophils, often with central necrosis or suppuration (5) (Figure 2). Older granulomas consist of a central necrosis surrounded by connective tissue and fewer inflammatory cells. Perivascular infiltration of macrophages is also a typical finding (5).

## Pathogenesis of Infection

The exact sequence of infection has not been clarified (23), but several studies indicate that the bacterium is able to penetrate through intact skin and gills followed by systemic invasion. Invasion through the oral and/or intestinal routes has also been suggested (24). To what extent the bacterium will survive passage through the stomach and the foregut is not known. Understanding infection routes are important for optimizing immunization protocols and will be discussed later.

The infection mechanisms at the cellular level are not understood in detail and different alternatives have been proposed: (i) the bacteria locate in cytoplasmic vacuoles in infected cells (18, 21), (ii) they are free in the cytoplasm, or (iii) reside outside cells (17). The localization in the intracellular compartment is tentative (18, 21) and has not been conclusively defined, and it is important as to what immune profile would be required for optimal protection. A recent study has shown that the bacterium is dependent on host cell clathrin for infection of macrophages, i.e., chloroquine treatment abolishes the infection (18). Further to this, results are indicative of the bacterium using actin through a disorganization process. Further, it seems that the bacterium induces *de novo* synthesis (of actin) to form vesicle in cytosolic compartments within which the bacterium resides (18) rather than using it for movement, as seen with *Listeria monocytogenes* (25). These responses could also facilitate export of the bacterium from the infected cells; however, this is more of a theory than actually proven experimentally (18). Further, there is also a possibility that the actin formation is involved in apoptosis induction in infected cells (26).

All referred studies have been carried out *in vitro* and translation to *in vivo* conditions carries some uncertainty but the cell types (SHK-1) used for *in vitro* studies derive from Atlantic salmon macrophages (18), a cell type that is infected by *P. salmonis in vivo* (21, 26, 27). To what extent compartmentalized localization of *P. salmonis* within vesicles would have a bearing on pathogenicity is not known but from a general viewpoint, it might play a role in immune evasion and likely also impact what immune mechanisms will be needed to obtain protective immunity, but this has not been studied in any detail for *P. salmonis*. Immune mechanisms and vaccination strategies will be discussed below.

Lipid A has also been implicated as playing a role in pathogenesis (3) and immunity. There has been speculation that the lipid A moiety of *P. salmonis* plays a role in disseminated intravascular coagulation of salmon (3), but this remains to be proven. It also seems somewhat speculative since salmonids do not express TLR-4 receptors (28) on any cell types. Further to this, salmonids are insensitive to LPS exposure when administered parentally (28, 29), aligned with the lack of TLR-4 receptors. Doses of 2–4 mg/ml of partly purified LPS originating from *Escherichia coli* or fish-pathogenic vibrios can be injected intraperitoneally (i.e., vaccinated) without treated individuals developing any signs of circulatory disturbance or clinical symptoms of shock (own observations). Even though it remains to be shown what role, if any, lipid A plays in pathogenesis of SRS and also for immune protection.

**Figure 3:**
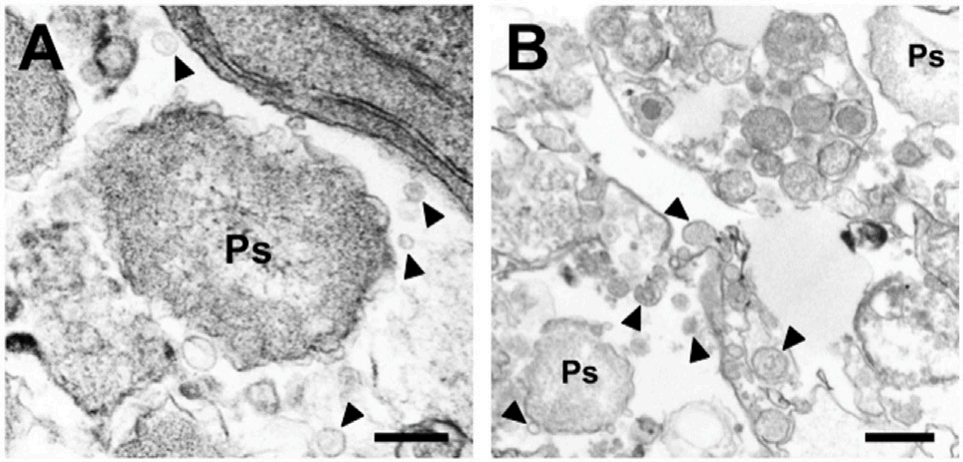
**Outer membrane vesicles of *P. salmonis* (Ps)**. Arrows point to membrane-coated vesicles (30). **(A)** Overview with bacterium (Ps). **(B)** Detail of vesicles (arrows).

More recently, it has been proposed that *P. salmonis* delivers some of its virulence factors *via* or by outer membrane vesicles (OMVs) to the infected cell (30). Similar OMV structures were also observed when the bacterium was grown in liquid media and HspP60 (heat shock protein) likely from the bacteria was found in these vesicles (30) (Figure 3).

## Spread of Infection

During natural infections, *P. salmonis* is transmitted horizontally from fish-to-fish without the need for physical contact (31, 32). No vector or intermediate host has been identified (17), and during experimental trials, the pathogen has been observed in the milt and celomic fluid of adult brood fish, and also in fry of infected brood fish, suggesting that the bacterium is transmitted vertically (33). *P. salmonis* has also been reported from outbreaks in freshwater (1), but is primarily a pathogen that causes clinical disease in sea water.

## Disease Prevention

Disease prevention strategies include reduced stress, improved husbandry practices (lower density, avoid transport/contact between farms, restrict movements of well-boats, fish, and people, separation of year-classes etc.), screening of brood stock, and vaccination. Current disease control practices also include use of antibiotics, since *P. salmonis* have been found susceptible to various antimicrobials. However, reduced sensitivity and increased resistance to penicillin, streptomycin, oxolinic acid, and oxytetracycline have been reported (12). There are commercial vaccines for intraperitoneal injection available, but these have shown variable results (34). Vaccinated fish come down with SRS toward the end of the production cycle which causes severe economic losses, i.e., death of fish close to harvest. In Chile, oral vaccines are used as a boost immunization the primary injection vaccination when the antibody titers decline (34). The different types of vaccines tested and those available commercially and their efficacy profiles are discussed below.

## Experimental Vaccines – Classical Inactivated Vaccines

Initial vaccination attempts to vaccinate against SRS were based on whole cell bacterins but with variable results (Table 2). Trials included vaccine preparations based on unconcentrated or concentrated preparations of formalin-killed *P. salmonis*, and these vaccine preparations gave contradictory results (35). Different inactivation methods were tested by Birkbeck et al. (36), and they administered a Scottish isolate of *P. salmonis* (SCO-95A) to Atlantic salmon by intraperitoneal injection, using either heat- or formalin-inactivated bacteria in adjuvant (oil-based adjuvant). They found that both vaccine types, heat- and formalin-inactivated provided significant protection against lethal challenge, RPS (relative percent survival)^1^ values of around 70 and 50%, respectively (36). Challenge was done at approximately 600 degree days (water temperature multiplied by number of days) post-vaccination and long-term protection was not assessed. The nature of the protective antigen was not identified or studied (19). These authors also studied the importance of the challenge conditions, and they found that experiments conducted at water temperatures of 7.5 to 8.5°C did not result in development of classical SRS, and no mortality was observed under these conditions. When experiments were run at 14°C, high level of mortality was achieved in control groups.

**Table 2:** **Summary of documented, immunoreactive antigens of *P. salmonis***.

Reference	Immunoreactive antigens of *Piscirickettsia salmonis*
Kuzyk et al. (71)	6 immunoreactive Ag (2 carbohydrates). Low humoral response in salmonids infected with these Ag
Barnes et al. (72)	6 immunoreactive Ag (2 carbohydrates, 4 proteins). One of the proteins homologous to Hsp60 of *Rickettsia tsutsugamushi*
Jones et al. (73)	9 protein bands and several non-protein bands were detected by immunoblot. Antigenic homogeneity observed among geographically diverse strains
Jamett et al. (74)	Developed and tested 6 monoclonal antibodies against *P. salmonis*
Marshall et al. (75)	Immunological characterization of Chaperon protein (membrane associated structural component)

## Experimental Vaccines – Subunit Vaccines

The use of subunit vaccines is dependent on the protective antigens being identified/known. When available, subunit vaccination strategies will make it possible to fine tune the vaccines to include only antigen(s) important for protection (Table 2). Kuzyk and coworkers (37) constructed an expression library from *P. salmonis* and cloned a 17-kDa outer surface lipoprotein (OspA) from the bacterium (Tables 2 and 3). This was used to immunize Coho salmon that developed strong antibody responses (38). Fish were subsequently challenged after being vaccinated with the recombinant OspA antigen and a high level of protection was obtained, RPS up to 83%. The protection improved when T cell epitopes from the tetanus toxin and measles virus fusion protein were included in the vaccine (38). This latter principle would skew the immune response in the direction of T-cell responses. These are the only studies published using this technology, and the vaccine concept has not been brought forward to a commercial product, the reason for which is not known but cost could be an issue.

**Table 3:** **Summary of published studies on vaccination studies, antigens used, and their obtained efficacy**.

Reference	Antigen	Vaccine type	Vaccine efficacy
Smith et al. (35)	Killed bacterin	Formalin inactivated	Inconsistent results
Kuzyk et al. (37)	OspA (outer surface protein A)	Recombinant subunit vaccine + T cell epitopes	83% RPS
Miquel et al. (48)	Whole genome	DNA (gene expression library)	Mortality 80% (inconsistent results)
Birkbeck et al. (36)	Inactivated bacterin (Scottish isolate SCO-95A)	Heat- or formalin-inactivated	70.7% RPS
			49.6% RPS
Wilhelm et al. (76)	Hsp60 Hsp70	Recombinant subunit vaccine	8% mortality
Salonius et al. (47) – Commercial vaccine against BKD (Canada)	*Arthrobacter davidanieli*	Live vaccine	Laboratory results 2% mortality. Field results 6.7% mortality
Wilhelm et al. (39)	(V1) Hsp60/70 + FlgG	Recombinant vaccine including Freund’s adjuvant	(V1) 95–94.5% RPS
	(V2) TbpB + MltB		(V2) 85%
	(V3) Omp27 + FlaA		(V3) 10.4%
Tobar et al. (56)	Inactivated bacterin, *P. salmonis* strain PS2C	Inactivated, whole bacterial antigen formulated in micromatrix for oral delivery or i.p. injection (or combined)	Onset of immunity by 300 degree days (oral group). IP boosted with oral (1500 degree days), good protection by 1800 degree days post primary immunization (300 degree days post boost)

In yet another study, Wilhelm et al. (39) elicited an immune response in Atlantic salmon following intraperitoneal injection of two heat shock proteins cloned from the bacterium, Hsp60 and Hsp70. Mixtures of Hsp60, Hsp70, and the flagellar protein (FlG) were used to immunize fish and this achieved an RPS of 95% (39). It was also shown that the antibody response persisted for 8 months or 2800 degree days post-vaccination (39), which is encouraging from a commercial standpoint. However, there are few if any follow-up studies based on these antigen combinations, and currently, there are no commercial vaccines available in the market based on the referred antigen combination(s) or other recombinant vaccine preparations. Cost of production is possibly one of the impediments.

## Immune Responses Induced from Inactivated/Subunit Vaccines

Inactivated vaccination protocols will in general elicit an immune response that is biased toward humoral immunity with lesser induction of cell-mediated immune responses (40), also seen in salmon (41). Antibodies will exert their biological effects through attachment to surface antigens of the pathogen. This will result in opsonization of bacteria, which again facilitates phagocytosis and intracellular killing by professional phagocytes (42) also well known in fish (43, 44). For viruses, neutralization prevents infection of target cells/organs and is an important mode of action (45). *P. salmonis* is an intracellular pathogen and a relevant question is to what extent circulating antibodies can prevent infection or aid in combating/limiting the spread of infection once established. While the exact pathological sequence is not understood for *P. salmonis* the antibodies could be beneficial during early stages of infection, from port of entry (gills, skin, and gut) and transport to primary or secondary multiplication sites. Then, could circulating antibodies play a role in limiting the spread of bacteria from cell to cell/organ to organ? This would require the bacterium to use an extracellular route of dissemination. It has not been determined in detail if *P. salmonis* spread from cell to cell *via* the extracellular space or use mechanisms similar to what has been described for *L. monocytogenes* that spreads directly from cell to cell, including *via* dying cells (46). If *P. salmonis* spreads through an extracellular route during early stages of infection, antibodies can be important for opsonization and subsequent killing in professional phagocytes. The fact that protection against disease is seen at early stages after sea transfer/early stage post-vaccination could favor an interpretation of spread through the extracellular space. Further to this, there are good indications of antibody consumption over time, i.e., decline in circulating levels of antibodies as a result of pathogen exposure/infection (34, 47). The approach has been to boost the primary response by oral antigen delivery with the purpose to raise the level of circulating antibodies. This will be discussed more below.

## Plasmid Vaccines and Replicating Vaccines

Given that humoral immune responses are insufficient in providing protection, a rational approach would be to explore vaccine modalities that elicit cell-mediated immune responses. Miquel and coworkers tested out a plasmid vaccine concept or a DNA vaccine (48). They used fragments of purified DNA from the bacterium based on an expression library that was cloned into the pCMV-Bios vector and subsequently used for immunization. They obtained two colony libraries corresponding to the genome of *P. salmonis*. Plasmid DNA was purified and administered by intramuscular injection into Coho salmon, which was followed by the second injection (plasmid-based boost) 40 days after primary immunization. Fish were challenged and only 20% of the vaccinated fish survived. The survivors had decreased bacterial load and the immune response was found specific to *P. salmonis* antigens with no cross-reaction to *Renibacterium salmoninarum* or *Yersinia ruckeri* (48). Such low level of protection would not be viable as a commercial product and there are currently no plasmid-based SRS vaccines available in the market.

There are no published studies documenting effect of live-attenuated vaccines, i.e., using attenuated strains of *P. salmonis*. There is one study based on immunization of salmon with live (replicating) *Arthrobacter davidanieli* that showed promising results in terms of increased survival in immunized and challenged Coho salmon compared to controls (47). This was also tested under field conditions with improved survival. The assumption is that the *Arthrobacter* species share antigens with *P. salmonis* and thus elicit cross reactive antibodies and/or immune effector T cells, but there are no published studies to support this notion. Attenuated vaccine strategies might be interesting alternatives to the inactivated vaccines given the nature of the infection (intracellular) and thus the need for cell-mediated immune responses (49). Recently, it was announced that a live-attenuated vaccine against SRS will be available in the Chilean market this year (50), but so far, there are no scientific reports to support the potential effect of the vaccine.

## Immune Responses Induced by DNA and Replicating Vaccines

**Figure 4:**
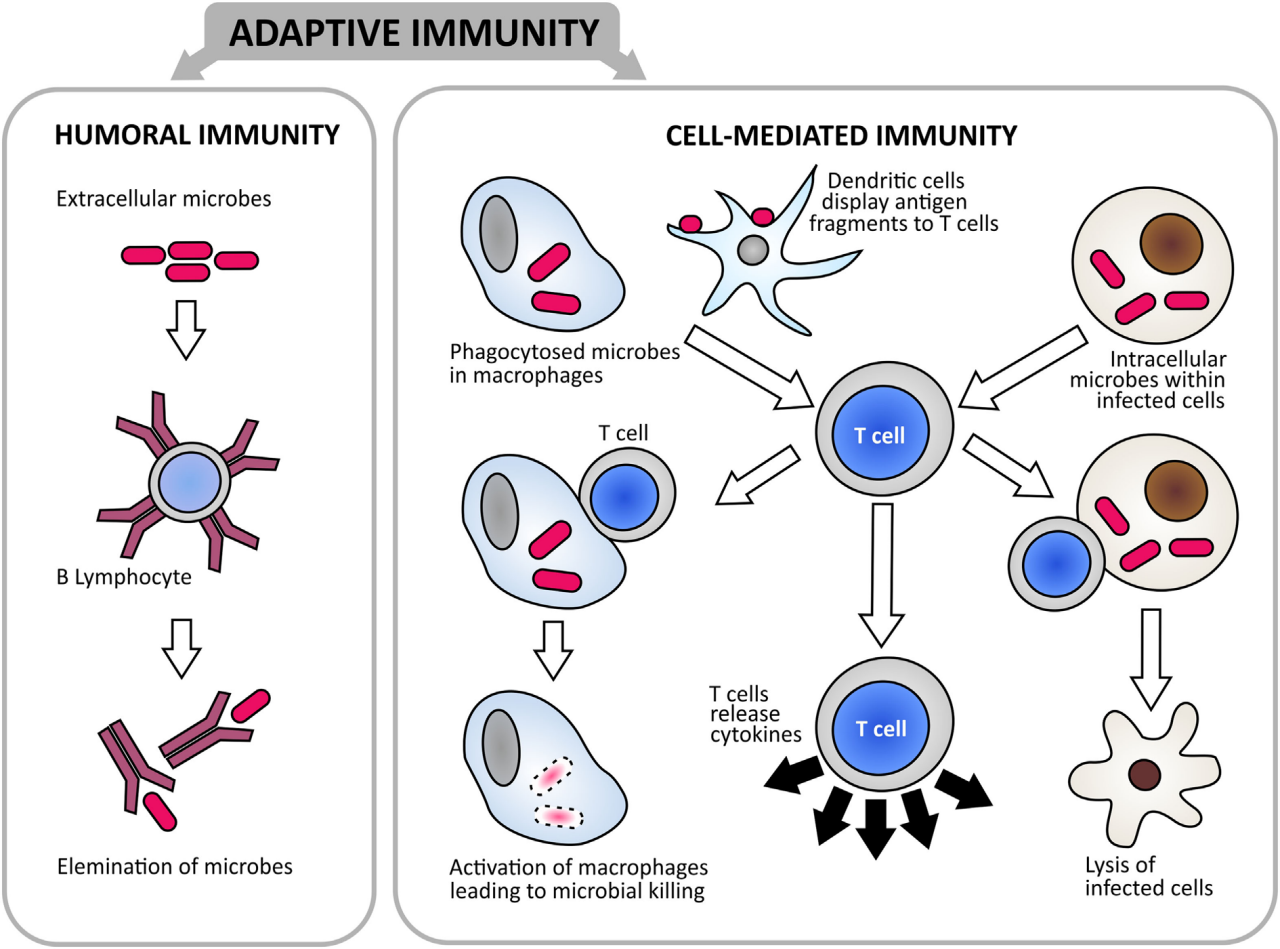
**There are two branches of adaptive immunity that constitute the protective mechanism against infectious diseases: humoral- and cell-mediated immunity**. Humoral immunity is mediated by antibodies that are produced by B cells. It is the main defense mechanism against extracellular pathogens, with secreted antibodies binding to pathogens and assist in their elimination. Cell-mediated immunity is mediated by T cells, with dendritic cells playing key roles in antigen presentation. T cells function by direct killing of cells infected with intracellular pathogens; activating macrophages to kill phagocytized pathogens; or by releasing cytokines to regulate the immune responses (65).

The immune responses elicited by DNA vaccines and replicating vaccines are biased toward cell-mediated immunity (49) (Figure 4). DNA vaccines elicit bias toward Th1 immunity, i.e., CD4 T cell activation. An attenuated vaccine would give a bias toward CD8 and T cytotoxic responses (49). It remains to be proven what immune responses actually play the most important role for preventing and/or controlling *P. salmonis* infection in salmonids. While there is no doubt that *P. salmonis* is an intracellular pathogen (27), it has not been fully documented whether the bacterium resides in endosomes/phagosomes [such as *Mycobacterium* sp. (51)] or is released from the initial endosome/phagosome to the cytosol, as seen for *L. monocytogenes* (52, 53). As was mentioned earlier, there are studies indicating that *P. salmonis* induces formation of vesicular structures from actin disorganization and *de novo* synthesis (18), but it is not known if this is also the site of bacterial growth/division and a source of spread to neighboring cells. The subcellular localization of the bacterium will impact on what type of immune responses would be required for induction of protective immunity. Translating the knowledge from *L. monocytogenes* vaccination studies, it is reasonable to assume that cell-mediated immunity, particularly cytotoxic immune responses, is needed (53) in addition to strong antibody responses.

## Commercial Vaccines

Currently, there are more than 25 different vaccines available in the Chilean market against SRS, extrapolating from a not so recent summary (54), when all combinations are counted or included. The market is dominated by whole cell vaccines (inactivated) and, currently, several vaccine combinations are available from Virbac-Centrovet (*n* = 12) and Pharmaq AS (*n* = 5) where both manufacturers provide vaccines for injection (multivalent vaccine for intraperitoneal delivery or monovalent vaccines). These vaccines are oil adjuvanted (water-in-oil emulsions) or live attenuated, and *P. salmonis* is combined with various/different bacterial antigens (for the inactivated vaccines). Other suppliers also sell commercial SRS vaccines in Chile. The experience is that the current vaccine concept confers good short-term protection against disease and mortality, but it has proven inefficient in conferring long-term protection, i.e., the duration of protection is not sufficient to protect the fish throughout their economic life. Virbac-Centrovet has also developed an oral vaccine against SRS designed for boosting after sea transfer (see below) and recently a live-attenuated vaccine became available for the Chilean market (Pharmaq).

## Translating Knowledge of Host–Pathogen Interactions into Optimized Vaccination Strategies

It is challenging to translate knowledge of host–pathogen interactions and detailed understanding of pathogenesis into improved vaccination strategies. This is particularly true when the pathogenic mechanisms are poorly or insufficiently understood, which to a large extent applies to *P. salmonis* infection in salmon. However, some of the recently gained knowledge of pathogenic events can potentially suggest alternative methods of vaccination/vaccination modalities.

**Figure 5:**
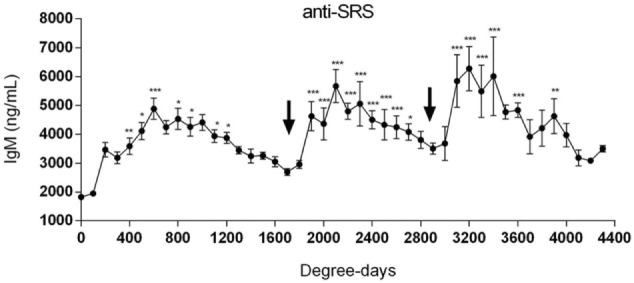
**Repeated oral immunizations to maintain a long-term protection against SRS**. Salmonids were primary immunized with an injectable mono or polyvalent vaccine against SRS. The arrows at 1700 and 2900 degree days indicate the time-point where first and second oral vaccines were administrated, respectively. Serum samples were obtained at different degree days to determine specific IgM anti-*P. salmonis*. Samples were statistically analyzed by one-way ANOVA – Dunnet post-test (150 fish/point) *p < 0.05; **p < 0.01; ***p < 0.001 (34).

From the above, it is fair to state that *P. salmonis* infects the fish not only through external surfaces mainly, i.e., skin and gills, but also through the gut mucosa although to a lesser extent (55). Current vaccination strategies based on non-replicating vaccines will elicit a humoral immune response and to a lesser extent cell-mediated immunity. The kinetics of the antibody response is sparsely studied, but recently, Tobar et al. (34, 56) showed that immunized fish had increased antibody levels up to 800 degree days post-vaccination after which they started to decline (Figure 5). There is a successive decline in antibody levels after they have peaked and they reach pre-vaccination levels by 1800–1900 degree days post-vaccination. The underlying mechanisms were not studied and are not known in detail but could be due to pathogen exposure and antibody consumption, as has been seen in IPN-vaccinated salmon (57). In line with such a thinking, the authors showed that declining levels of anti-*P. salmonis* antibodies coincide with increase in SRS-related mortality (34) indicating that pathogen exposure at least partly explained antibody consumption. Further, oral revaccination increased the circulating antibodies to levels equivalent to or higher than what was achieved from the primary injection vaccine (34), and even more pronounced when a second oral boost was administered (Figure 5). This study is one, among few, showing the circulating antibody levels in salmon show a boost pattern when primary immunization is by the parenteral route (intraperitoneal) and boost by oral delivery. Previously, this has also been shown for IPN vaccines in salmon (58), and it should be added that for both these studies, IgM was the immunoglobulin isotype measured in serum. Underpinning these observations is a recent study on prime-boost vaccination against infectious salmon anemia virus where the same response pattern was observed (34). Further, the effect of repeated oral boost has recently been shown for feed-based vaccination of red tilapia (*Oreochromis niloticus x Oreochromis mossambicus*) against streptococcosis (59). In the referred studies, no attempts were made to measure IgT on mucosal surfaces, but in the study by Chen et al. (60) transcript levels of IgT in the gut mucosa was measured and increased mRNA expression levels were found. The functional importance is, however, not known. From these studies, the proposed rationale to boost primary immunization by repeated oral boost (34) seems like a relevant and good proposition, but it remains to be documented from additional field studies. The mechanisms of protection against mortality are not fully understood, although the authors observed that protection against disease coincided with level of antibodies. As has been discussed above, it is not obvious that circulating antibodies will protect against infection or disease development, for the mere reason that *P. salmonis* has an intracellular infection and multiplication strategy. That said, impeding infection efficiency at site(s) of entry, such as gills and skin, and also gut, could play a role in limiting infection success, and it is conceivable that oral boosting will also result in production of IgT in the mucosal lining. It is well known that infection can result in formation of pathogen specific IgT at mucosal surfaces (61–63). However, to what extent antibodies (IgT) are formed at mucosal surfaces following vaccination and to what extent they actually protect against infection or merely contribute to regulating the commensal flora as has been shown in higher vertebrates (64) or both, is not fully understood. Nevertheless, the reasoning that injection vaccines (one injection) confer too short protection against mortality (or infection), and applying a concept where fish are monitored for their antibody levels post-vaccination (34) and then boosted is a good rationale. This concept needs to be further explored, not only for *P. salmonis* infection but also for other diseases/pathogens.

## Author Contributions

The author confirms being the sole contributor of this work and approved it for publication.

## Conflict of Interest Statement

The author declares that the research was conducted in the absence of any commercial or financial relationships that could be construed as a potential conflict of interest.

## Footnotes

^1^Relative percent survival is calculated on the basis of the formula; RPS = [1 − (mortality in controls/mortality in vaccinates) × 100].
